# Dormancy Leading to Late Recurrence in Breast Cancer: A Case of Hormone Receptor-Positive Supraclavicular Metastasis 10 Years After the Initial Treatment

**DOI:** 10.7759/cureus.71586

**Published:** 2024-10-16

**Authors:** Mena Louis, Rafael Tapia, Nathaniel Grabill, Priscilla Strom

**Affiliations:** 1 General Surgery, Northeast Georgia Medical Center Gainesville, Gainesville, USA; 2 Surgery, Northeast Georgia Medical Center Gainesville, Gainesville, USA

**Keywords:** breast cancer metastasis, dormant tumor cells, hormone receptor-positive, late recurrence breast cancer, supraclavicular lymph nodes

## Abstract

Breast cancer recurrence can occur many years after the initial treatment, particularly in hormone receptor-positive (HR+) cases, where the risk of late recurrence remains significant. Late recurrences are well documented, with research showing that they can happen even decades after the primary diagnosis, necessitating extended monitoring and personalized therapeutic approaches.

A 65-year-old woman with a history of stage IIIC invasive ductal carcinoma, initially treated with neoadjuvant chemotherapy, bilateral mastectomies, adjuvant chemoradiation, and prolonged hormonal therapy, presented 10 years later with metastasis to the left supraclavicular lymph nodes. A biopsy confirmed recurrent ER+/PR+/HER2- (estrogen receptor-positive/progesterone receptor-positive/human epidermal growth factor receptor 2-negative) breast cancer. Her treatment was adjusted to include Faslodex (fulvestrant) and Verzenio (abemaciclib), followed by the surgical resection of the metastatic lymph node.

Managing HR+ breast cancer involves significant challenges, mainly due to the potential for late recurrence. Even after aggressive treatment and years of remission, dormant tumor cells may become active again, leading to metastasis in less common sites, like the supraclavicular lymph nodes. This situation demands a tailored therapeutic approach, adjusting treatment strategies to address the specific characteristics of the recurrent tumor.

In conclusion, late recurrence in HR+ breast cancer requires vigilant long-term follow-up and personalized treatments to effectively manage recurrence risk. Understanding dormancy and reactivation mechanisms is essential for guiding clinical decisions. Prioritizing individualized follow-up strategies and refining treatment protocols will be key to improving patient outcomes and maintaining quality of life.

## Introduction

Breast cancer is a major global health concern, with the potential for recurrence even after years of remission [1]. Advances in early detection and treatment have significantly improved survival rates [2]. However, the occurrence of late recurrence, particularly in hormone receptor-positive (HR+) cases, continues to pose difficulties in both monitoring and treatment [3]. Such recurrences are often due to dormant cancer cells that may become active again long after the initial therapy [4].

Understanding the mechanisms behind breast cancer dormancy and late recurrence has become a critical focus in oncology [5]. Research has highlighted that specific breast cancer subtypes, particularly those that are estrogen receptor-positive (ER+), are prone to late recurrences [4,6]. These recurrences can occur decades after the primary treatment, often manifesting in regional lymph nodes or distant organs [7]. This latency requires clinicians to maintain vigilance and consider long-term follow-up strategies, including extended endocrine therapy, to mitigate the risk of recurrence [6].

The clinical management of late breast cancer recurrence involves a multidisciplinary approach, integrating surgical, medical, and radiation oncology [8]. The therapeutic strategies must be adapted to the unique biological characteristics of the recurrent tumor, which may differ from those of the original primary tumor [9]. Personalized treatment plans are essential to optimize outcomes, especially in the context of advanced and targeted therapies that have evolved in recent years [10].

## Case presentation

A 65-year-old woman with a history of stage IIIC (T2 N3a M0) invasive ductal carcinoma of the left breast presented with a new left supraclavicular mass. Her initial diagnosis involved a 5.0 x 3.4 cm subareolar mass with extensive axillary lymph node involvement, confirmed by bilateral breast magnetic resonance imaging (MRI) and ultrasound-guided core biopsy. The tumor was characterized as ER+ at 92%, progesterone receptor positive (PR+) at 63%, and human epidermal growth factor receptor 2 (HER2)/neu negative, with a high proliferation index (Ki-67 of 92%). A staging workup, including positron emission tomography-computed tomography (PET-CT), showed no evidence of distant metastasis. She received neoadjuvant chemotherapy with FEC (fluorouracil, epirubicin, cyclophosphamide) followed by Taxol, leading to tumor size reduction.

Subsequent treatment included a left modified radical mastectomy and a right simple mastectomy with immediate implant reconstruction. Pathology revealed a residual 1.9 cm tumor, with 16 out of 17 axillary lymph nodes positive for metastatic disease. Postoperatively, she underwent two cycles of adjuvant chemotherapy with Gemzar (gemcitabine) and carboplatin, followed by radiation therapy. She was maintained on Arimidex (anastrozole) as part of her adjuvant hormonal therapy until she detected a nodularity in the left supraclavicular region.

**Figure 1:**
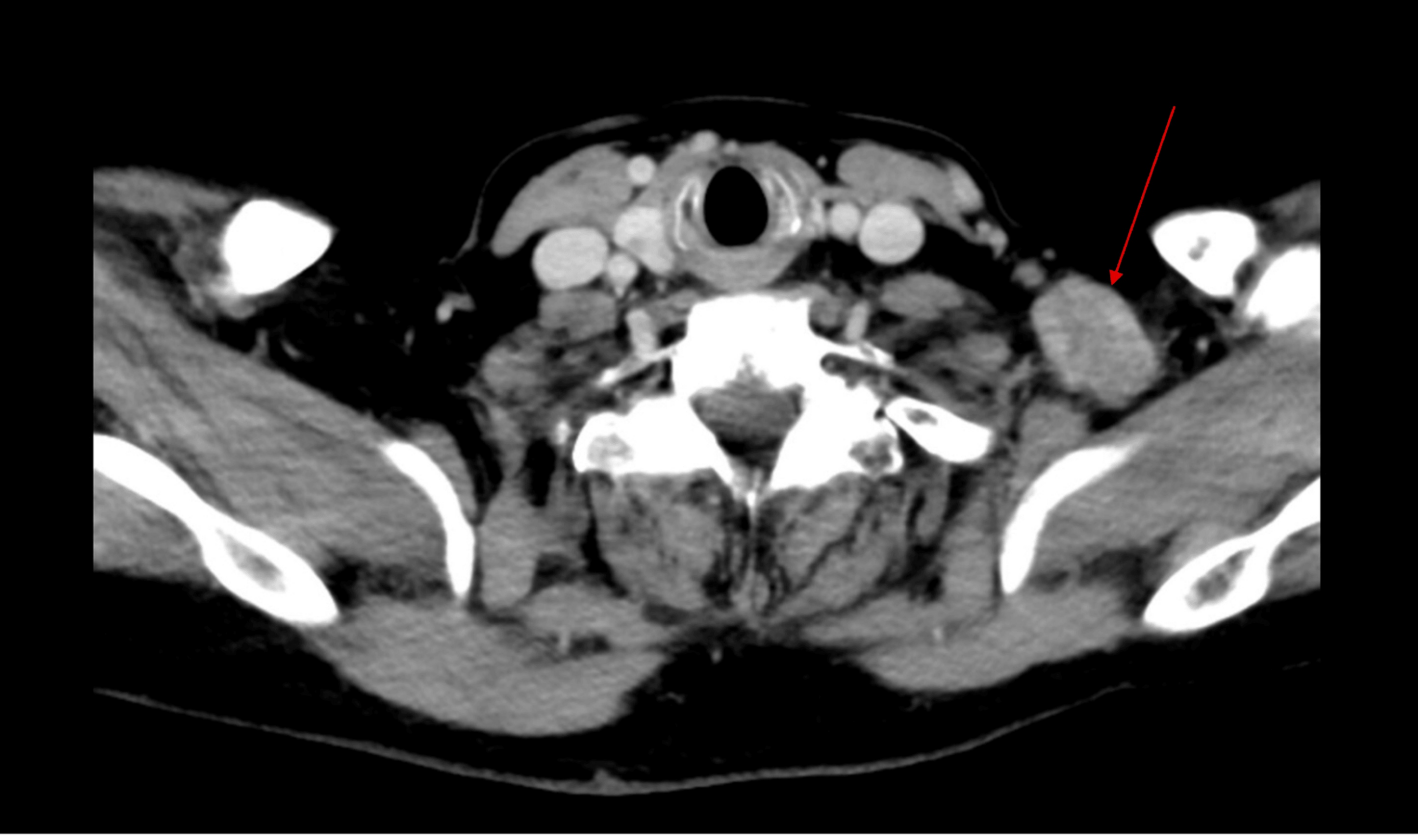
Axial view of a CT soft tissue neck with contrast, highlighting an enlarged left supraclavicular lymph node indicated by a red arrow. CT: computed tomography

**Figure 2:**
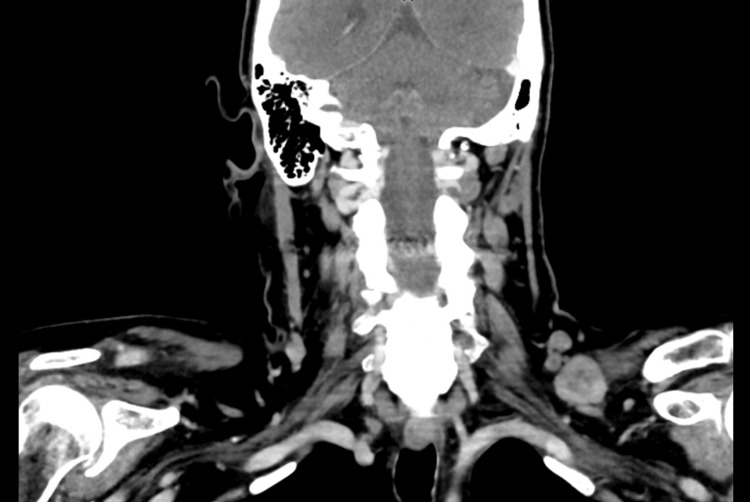
CT soft tissue neck with IV contrast, showing an enlarged left supraclavicular lymph node within a red square, measuring approximately 3.2 x 2.3 cm. CT: computed tomography

**Figure 3:**
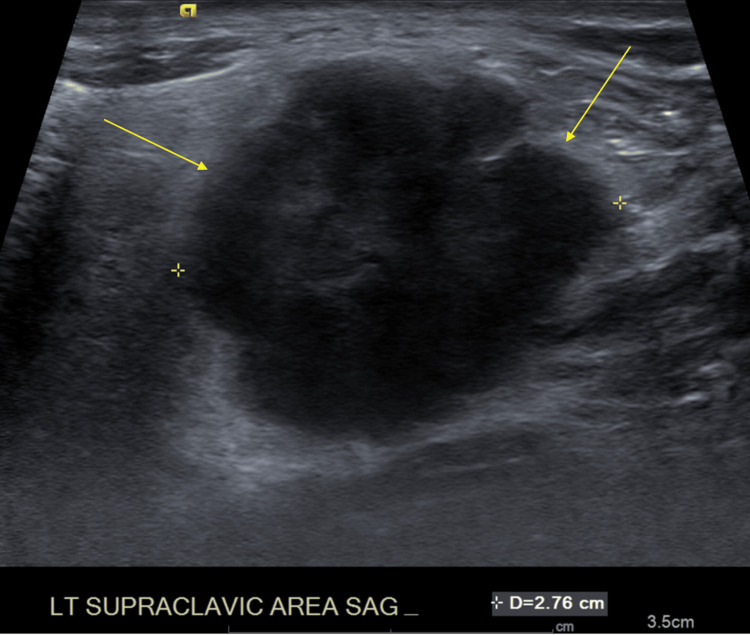
Ultrasound of the left supraclavicular region, highlighting lymphadenopathy marked by yellow arrows.

**Figure 4:**
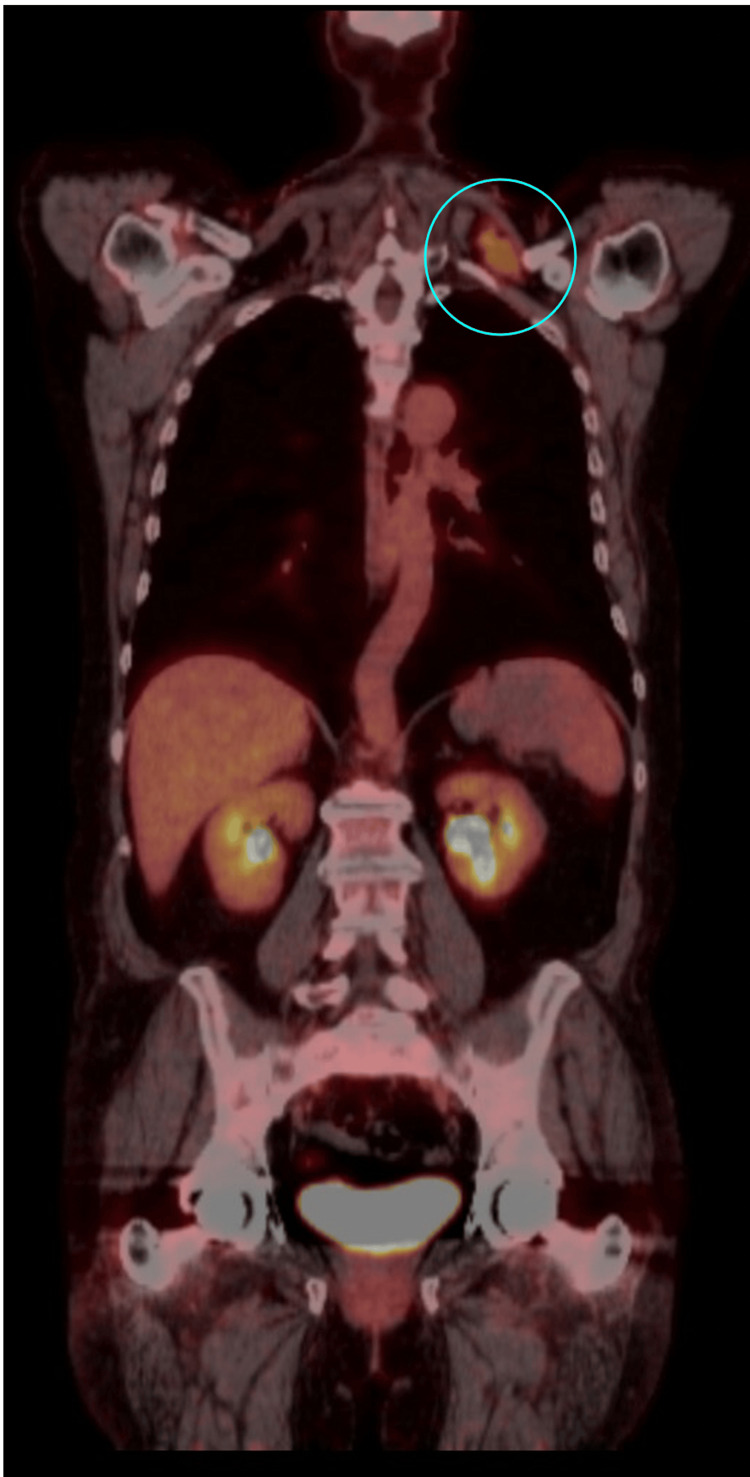
PET-CT scan from skull to thigh, coronal view, with a cyan circle marking a solitary FDG-avid left supraclavicular lymph node, measuring 3.6 x 2.4 cm with an SUV max of 4.71. No other suspicious FDG-avid lesions are observed. PET-CT: positron emission tomography-computed tomography; FDG: fluorodeoxyglucose; SUV: standardized uptake value

**Figure 5:**
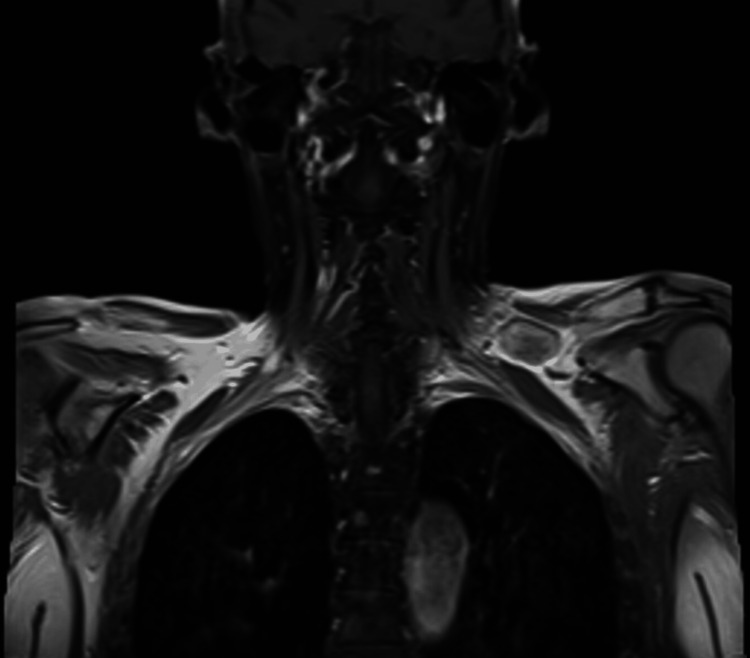
MRI of the brachial plexus, with a purple arrow indicating left supraclavicular adenopathy, showing no brachial plexus involvement. MRI: magnetic resonance imaging

Ten years later, the patient presented with a palpable left supraclavicular mass. A CT scan of the neck and chest revealed left supraclavicular adenopathy, with the largest lymph node measuring 3.3 x 2.3 cm (Figures 1-2). An ultrasound-guided core biopsy confirmed recurrent metastatic breast cancer, which remained ER+ at 90%, PR+ at 52%, and HER2 1+ (negative) (Figure 3). A PET-CT scan identified a solitary fluorodeoxyglucose (FDG)-avid lesion in the left supraclavicular region, measuring 3.6 x 2.4 cm with a standardized uptake value (SUV) max of 4.7, without evidence of other FDG-avid disease (Figure 4). An MRI of the brachial plexus showed no evidence of involvement (Figure 5). She subsequently underwent resection of the left neck mass, with pathology confirming metastatic carcinoma consistent with breast primary in all six lymph nodes and significant extracapsular extension.

In response to the recurrence, her hormonal therapy was adjusted, discontinuing Arimidex and initiating treatment with Faslodex (fulvestrant) and Verzenio (abemaciclib). The patient tolerated the surgery well and continues to be closely monitored with ongoing systemic therapy.

## Discussion

Breast cancer metastasis can indeed occur many years after the initial treatment for the primary tumor, a phenomenon well-known as 'late recurrence' [11,12]. This is particularly associated with HR+ breast cancers, where the risk of late recurrence remains substantial even decades after the initial diagnosis [6,7]. Studies, such as those from the Danish Breast Cancer Group, have shown that recurrences can continue up to 32 years after primary treatment, especially in patients with larger tumor sizes, positive lymph nodes, and ER+ tumors [5,13]. Effective patient care may require long-term monitoring and possibly more prolonged treatment options [8].

In this case, the late recurrence presented as metastasis to the left supraclavicular lymph nodes, a rare but recognized pattern of spread in breast cancer [14]. Supraclavicular metastasis years after the initial treatment suggests the presence of dormant tumor cells that remained inactive before reactivating, possibly due to changes in the microenvironment or other systemic factors [15]. Such dormant cells are a significant concern in HR+ breast cancers, as they are known to have the potential for late reactivation and metastasis [16]. The reappearance of cancer in the supraclavicular region often signals more extensive disease involvement, necessitating thorough restaging and tailored treatment approaches [17,18].

Long-term follow-up in breast cancer survivors, especially those with HR+ tumors, is crucial due to the unique risks posed by late recurrences. Even though late recurrences may tend to be less aggressive compared to early recurrences, they still carry significant clinical implications. Dormant cancer cells, often associated with HR+ tumors, may remain inactive for many years after treatment, only to reactivate under certain conditions. This process, known as tumor dormancy, highlights the unpredictable nature of breast cancer recurrence. Studies have demonstrated that late recurrences, particularly in HR+ breast cancer, can occur even decades after initial treatment. Given this, vigilant monitoring is essential - not only to detect any signs of recurrence early, but also to manage any potential changes in the biological behavior of the tumor over time [9,10]. The reactivation of dormant cancer cells, particularly in HR+ breast cancer, is influenced by several factors. Changes in the tumor microenvironment, such as inflammation or angiogenesis, can disrupt the balance of dormancy, while immune system evasion mechanisms may allow dormant cells to escape immune surveillance. Hormonal fluctuations, especially in HR+ tumors, can also play a significant role in reactivating these cells, as can cellular stressors like oxidative damage or chemotherapy-induced DNA damage. Collectively, these factors create conditions that may push dormant cells back into active proliferation, contributing to late recurrence.

In managing these patients, tailored strategies must be employed, considering both the individual patient’s risk profile and the characteristics of their initial tumor [5]. Advances in imaging technologies, molecular testing, and biomarkers have improved the ability to predict the likelihood of recurrence, helping to identify those at higher risk [11]. Additionally, the introduction of extended adjuvant therapies, such as prolonged hormonal therapy for HR+ tumors, has demonstrated benefits in reducing the risk of late recurrence. However, these treatments come with potential side effects, necessitating a careful balance between the benefits of extended therapy and the patient’s quality of life [12]. Ultimately, the goal is to ensure that breast cancer survivors receive optimal care throughout their survivorship, with a focus on preventing and managing recurrences over the long term.

The management of this patient required adapting the therapeutic strategy to address the recurrent disease's biological characteristics, which remained ER+ but had a slightly reduced PR expression. The transition from Arimidex (anastrozole) to a combination of Faslodex (fulvestrant) and Verzenio (abemaciclib) is a critical therapeutic shift in this case, reflecting an advanced, personalized approach to managing HR+ breast cancer. While Arimidex effectively suppresses estrogen production, extended use may not completely prevent recurrence, particularly in high-risk patients with dormant cancer cells that can reactivate. By adding Faslodex, which degrades the ER, and Verzenio, a cyclin-dependent kinases 4/6 (CDK4/6) inhibitor that targets the cell cycle, this treatment strategy goes beyond hormonal control to directly inhibit cancer cell proliferation. This dual-target approach tackles both the hormonal and proliferative pathways of recurrent HR+ tumors, providing a more comprehensive solution for controlling disease progression. The choice of surgical resection for the solitary supraclavicular mass reflects an attempt to achieve local control, which is particularly important in isolated recurrences [8].

## Conclusions

Late recurrence in breast cancer, particularly in HR+ subtypes, presents a significant challenge in long-term patient management. The persistence and eventual reactivation of dormant cancer cells can lead to metastasis many years after the initial treatment, necessitating extended follow-up and tailored therapeutic strategies. Understanding the biological mechanisms behind these late recurrences, as well as implementing vigilant surveillance, are crucial for improving outcomes and managing the risks associated with long-term cancer dormancy.
